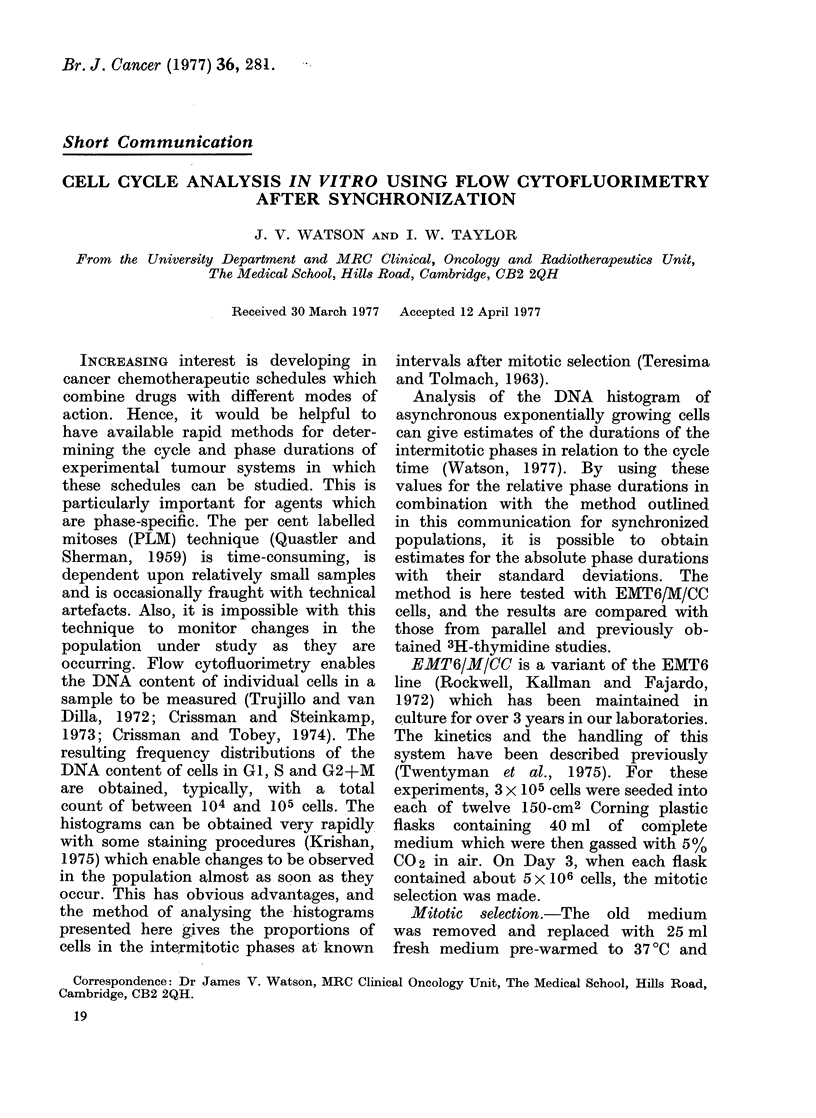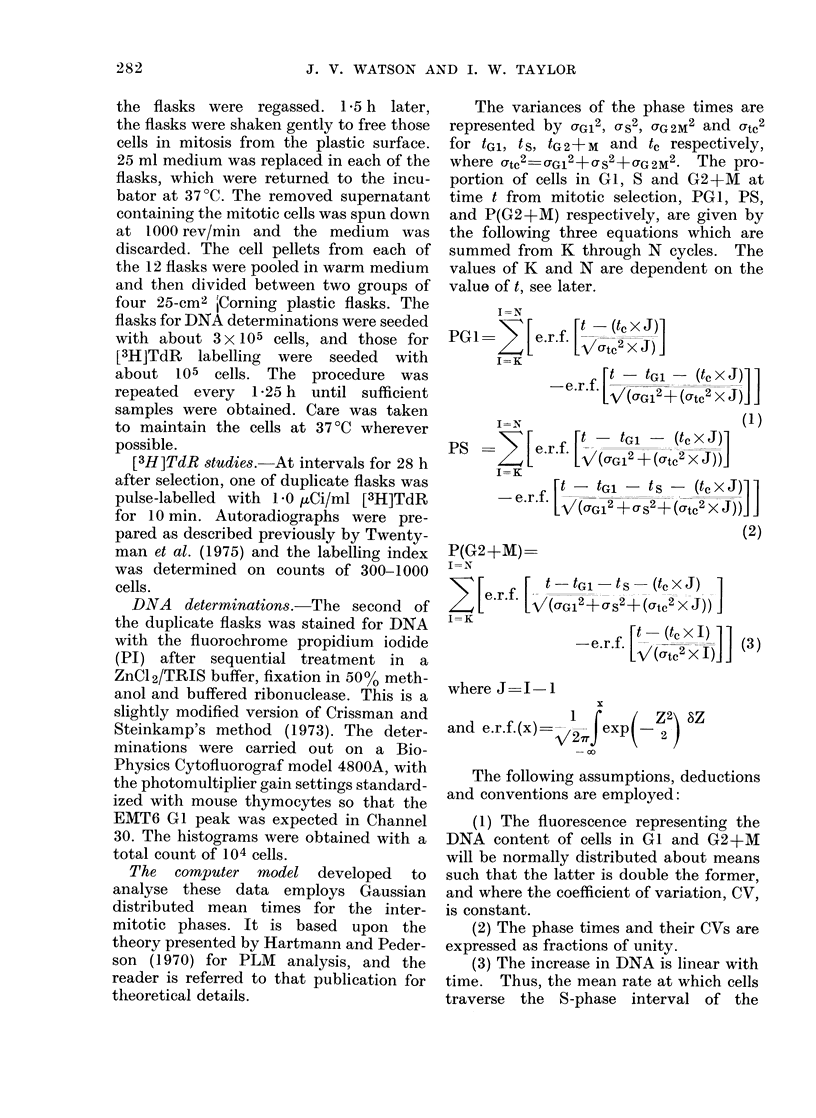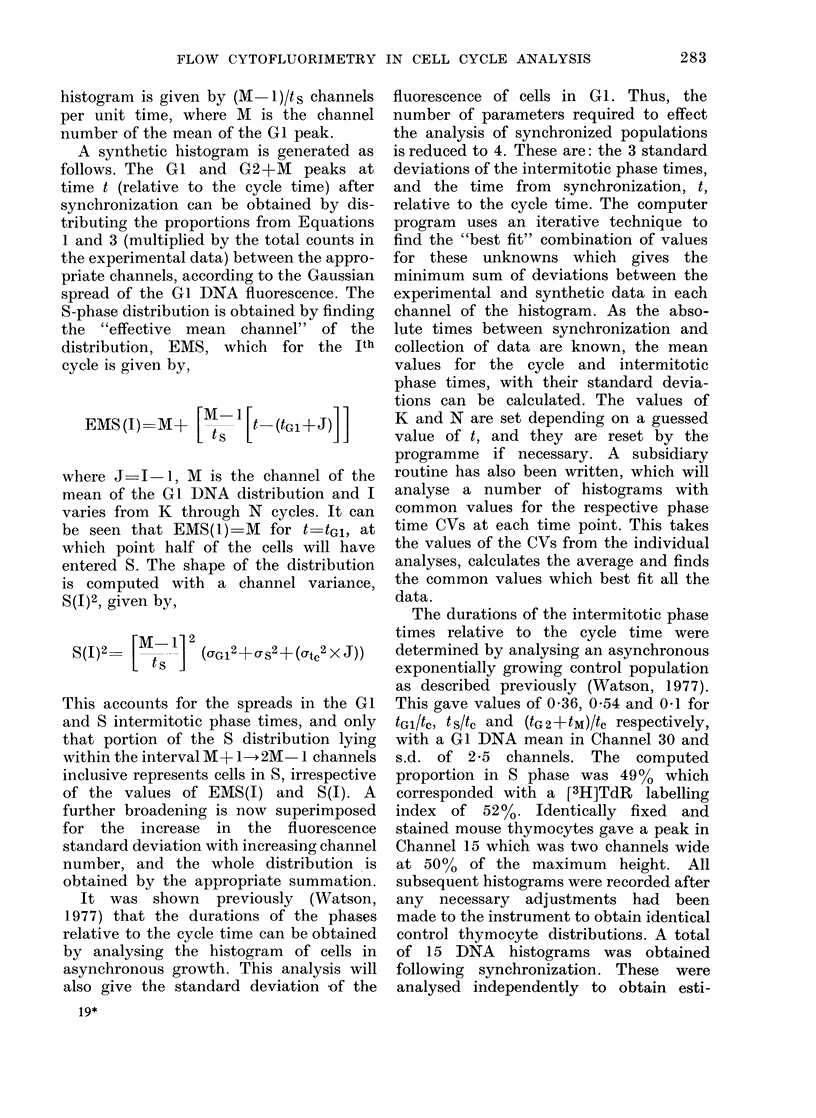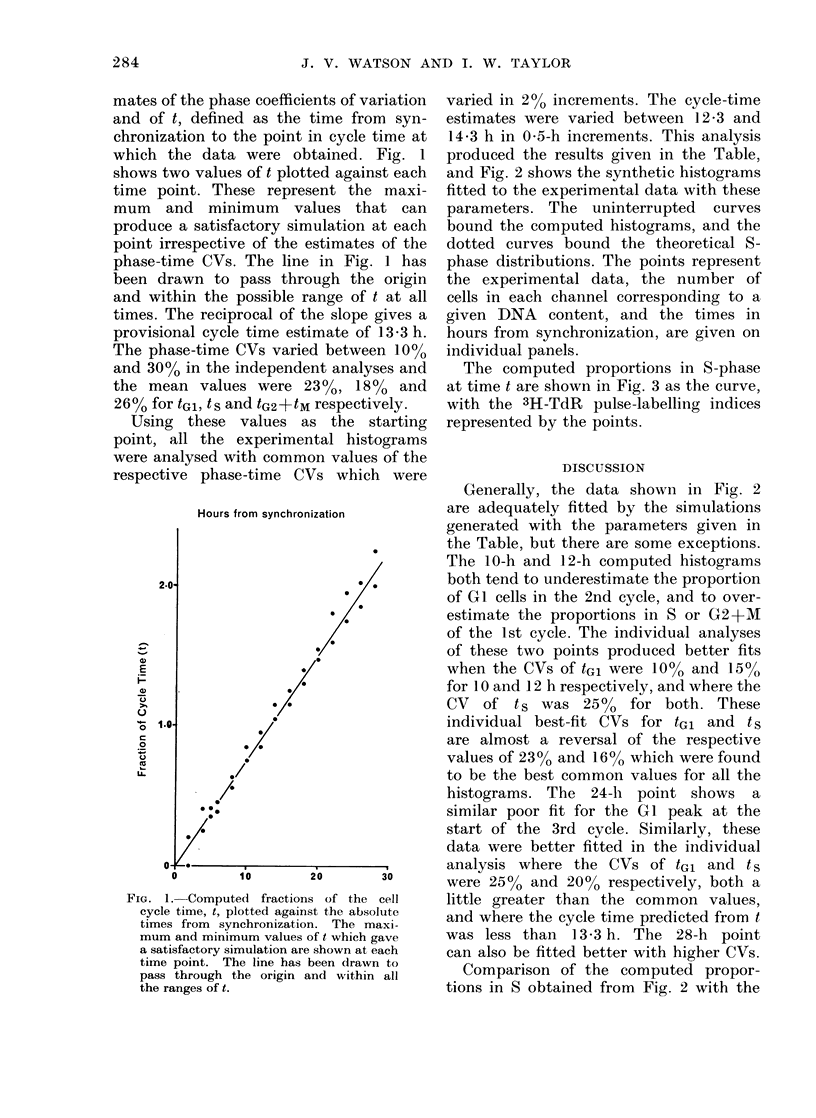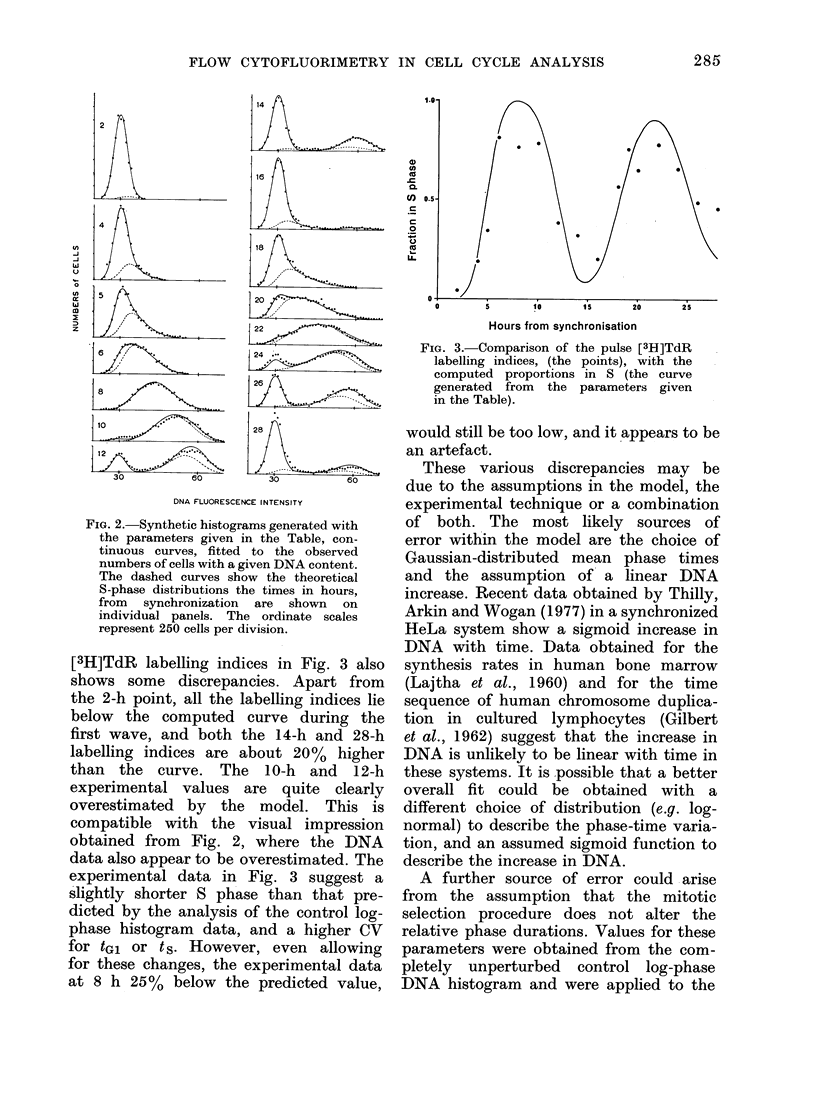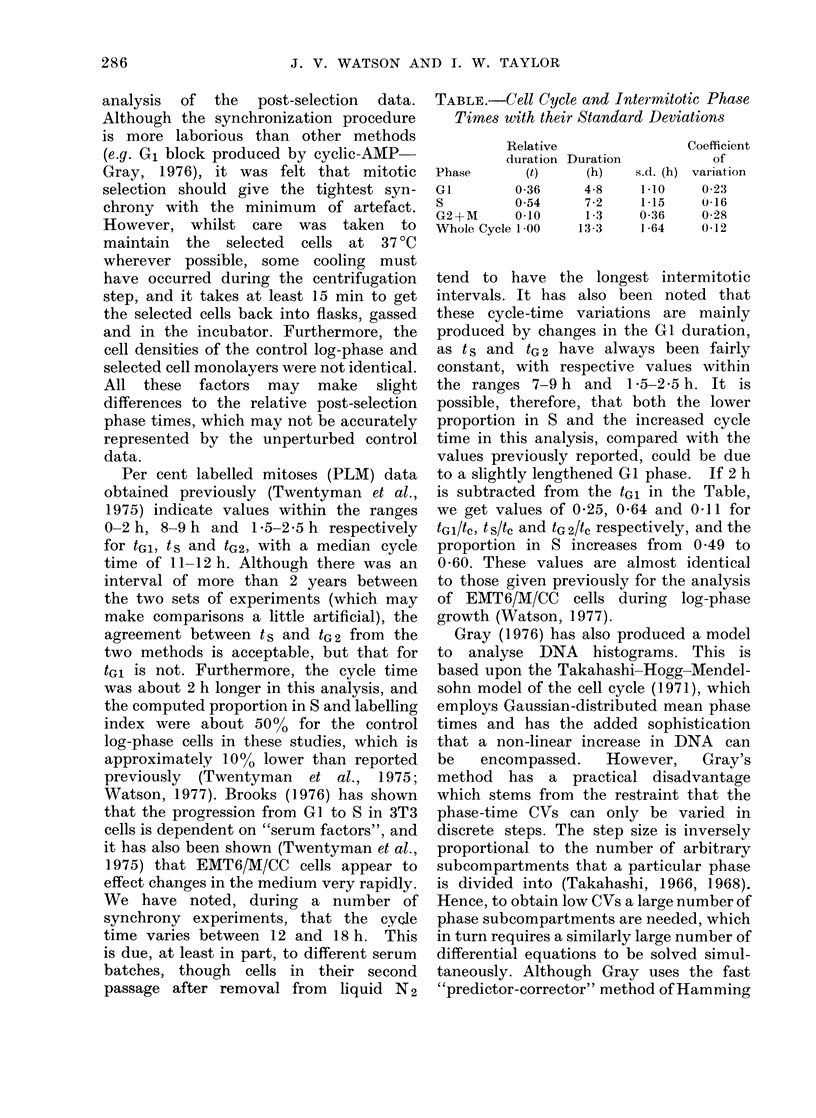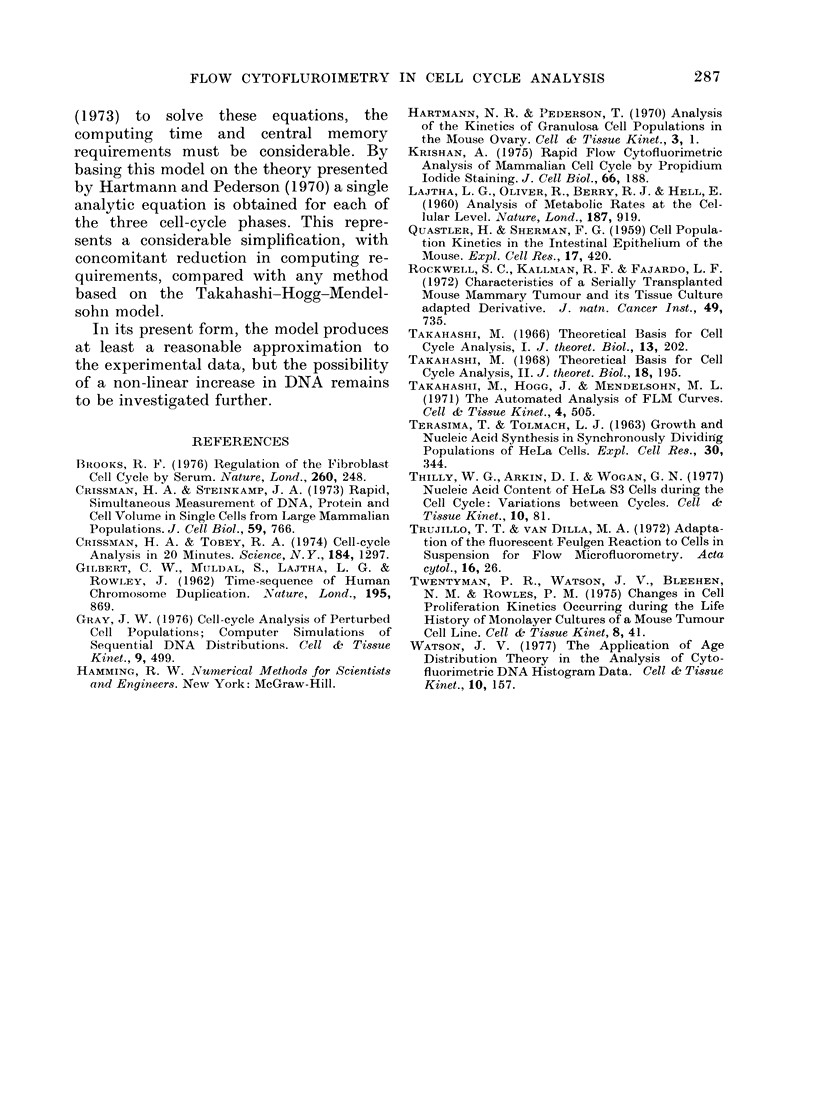# Cell cycle analysis in vitro using flow cytofluorimetry after synchronization.

**DOI:** 10.1038/bjc.1977.188

**Published:** 1977-08

**Authors:** J. V. Watson, I. W. Taylor


*Br. J. Cancer* (1977) **36**, 281.

### *Short Communication*

### CELL CYCLE ANALYSIS *IN VITRO* USING FLOW CYTOFLUORIMETRY AFTER SYNCHRONIZATION

J. V. WATSON AND I. W. TAYLOR

*From the University Department and MRC Clinical, Oncology and Radiotherapeutics Unit, The Medical School, Hills Road, Cambridge, CB2 2QH*

Received 30 March 1977 Accepted 12 April 1977

INCREASING interest is developing in cancer chemotherapeutic schedules which combine drugs with different modes of action. Hence, it would be helpful to have available rapid methods for determining the cycle and phase durations of experimental tumour systems in which these schedules can be studied. This is particularly important for agents which are phase-specific. The per cent labelled mitoses (PLM) technique (Quastler and Sherman, 1959) is time-consuming, is dependent upon relatively small samples and is occasionally fraught with technical artefacts. Also, it is impossible with this technique to monitor changes in the population under study as they are occurring. Flow cytofluorimetry enables the DNA content of individual cells in a sample to be measured (Trujillo and van Dilla, 1972; Crissman and Steinkamp, 1973; Crissman and Tobey, 1974). The resulting frequency distributions of the DNA content of cells in G1, S and G2+M are obtained, typically, with a total count of between $10^4$ and $10^5$ cells. The histograms can be obtained very rapidly with some staining procedures (Krishan, 1975) which enable changes to be observed in the population almost as soon as they occur. This has obvious advantages, and the method of analysing the histograms presented here gives the proportions of cells in the intermitotic phases at known intervals after mitotic selection (Teresima and Tolmach, 1963).

Analysis of the DNA histogram of asynchronous exponentially growing cells can give estimates of the durations of the intermitotic phases in relation to the cycle time (Watson, 1977). By using these values for the relative phase durations in combination with the method outlined in this communication for synchronized populations, it is possible to obtain estimates for the absolute phase durations with their standard deviations. The method is here tested with EMT6/M/CC cells, and the results are compared with those from parallel and previously obtained $^3$H-thymidine studies.

*EMT6/M/CC* is a variant of the EMT6 line (Rockwell, Kallman and Fajardo, 1972) which has been maintained in culture for over 3 years in our laboratories. The kinetics and the handling of this system have been described previously (Twentyman *et al.*, 1975). For these experiments, $3 \times 10^5$ cells were seeded into each of twelve 150-cm$^2$ Corning plastic flasks containing 40 ml of complete medium which were then gassed with 5% $CO_2$ in air. On Day 3, when each flask contained about $5 \times 10^6$ cells, the mitotic selection was made.

*Mitotic selection*.—The old medium was removed and replaced with 25 ml fresh medium pre-warmed to 37 °C and

Correspondence: Dr James V. Watson, MRC Clinical Oncology Unit, The Medical School, Hills Road, Cambridge, CB2 2QH.

the flasks were regassed. 1·5 h later, the flasks were shaken gently to free those cells in mitosis from the plastic surface. 25 ml medium was replaced in each of the flasks, which were returned to the incubator at 37 °C. The removed supernatant containing the mitotic cells was spun down at 1000 rev/min and the medium was discarded. The cell pellets from each of the 12 flasks were pooled in warm medium and then divided between two groups of four 25-cm$^2$ Corning plastic flasks. The flasks for DNA determinations were seeded with about $3\times10^5$ cells, and those for [$^3$H]TdR labelling were seeded with about $10^5$ cells. The procedure was repeated every 1·25 h until sufficient samples were obtained. Care was taken to maintain the cells at 37 °C wherever possible.

*[$^3$H]TdR studies.*—At intervals for 28 h after selection, one of duplicate flasks was pulse-labelled with 1·0 $\mu$Ci/ml [$^3$H]TdR for 10 min. Autoradiographs were prepared as described previously by Twentyman *et al.* (1975) and the labelling index was determined on counts of 300–1000 cells.

*DNA determinations.*—The second of the duplicate flasks was stained for DNA with the fluorochrome propidium iodide (PI) after sequential treatment in a $ZnCl_2$/TRIS buffer, fixation in 50% methanol and buffered ribonuclease. This is a slightly modified version of Crissman and Steinkamp's method (1973). The determinations were carried out on a Bio-Physics Cytofluorograf model 4800A, with the photomultiplier gain settings standardized with mouse thymocytes so that the EMT6 G1 peak was expected in Channel 30. The histograms were obtained with a total count of $10^4$ cells.

*The computer model* developed to analyse these data employs Gaussian distributed mean times for the intermitotic phases. It is based upon the theory presented by Hartmann and Pederson (1970) for PLM analysis, and the reader is referred to that publication for theoretical details.

The variances of the phase times are represented by $\sigma_{G1}^2$, $\sigma_S^2$, $\sigma_{G2M}^2$ and $\sigma_{tc}^2$ for $t_{G1}$, $t_S$, $t_{G2+M}$ and $t_c$ respectively, where $\sigma_{tc}^2=\sigma_{G1}^2+\sigma_S^2+\sigma_{G2M}^2$. The proportion of cells in G1, S and G2+M at time $t$ from mitotic selection, PG1, PS, and P(G2+M) respectively, are given by the following three equations which are summed from K through N cycles. The values of K and N are dependent on the value of $t$, see later.

$$\mathrm{PG1}=\sum_{I=K}^{I=N}\left[\mathrm{e.r.f.}\left[\frac{t-(t_c\times J)}{\sqrt{\sigma_{tc}^2\times J)}}\right] - \mathrm{e.r.f.}\left[\frac{t-t_{G1}-(t_c\times J)}{\sqrt{(\sigma_{G1}^2+(\sigma_{tc}^2\times J)}}\right]\right] \quad (1)$$

$$\mathrm{PS}=\sum_{I=K}^{I=N}\left[\mathrm{e.r.f.}\left[\frac{t-t_{G1}-(t_c\times J)}{\sqrt{(\sigma_{G1}^2+(\sigma_{tc}^2\times J))}}\right] - \mathrm{e.r.f.}\left[\frac{t-t_{G1}-t_S-(t_c\times J)}{\sqrt{(\sigma_{G1}^2+\sigma_S^2+(\sigma_{tc}^2\times J))}}\right]\right] \quad (2)$$

$$\mathrm{P(G2+M)}=\sum_{I=K}^{I=N}\left[\mathrm{e.r.f.}\left[\frac{t-t_{G1}-t_S-(t_c\times J)}{\sqrt{(\sigma_{G1}^2+\sigma_S^2+(\sigma_{tc}^2\times J))}}\right] - \mathrm{e.r.f.}\left[\frac{t-(t_c\times I)}{\sqrt{(\sigma_{tc}^2\times I)}}\right]\right] \quad (3)$$

where $J=I-1$

$$\text{and e.r.f.}(x)=\frac{1}{\sqrt{2\pi}}\int_{-\infty}^{x}\exp\left(-\frac{Z^2}{2}\right)\delta Z$$

The following assumptions, deductions and conventions are employed:

(1) The fluorescence representing the DNA content of cells in G1 and G2+M will be normally distributed about means such that the latter is double the former, and where the coefficient of variation, CV, is constant.

(2) The phase times and their CVs are expressed as fractions of unity.

(3) The increase in DNA is linear with time. Thus, the mean rate at which cells traverse the S-phase interval of the histogram is given by $(M-1)/t_S$ channels per unit time, where M is the channel number of the mean of the G1 peak.

A synthetic histogram is generated as follows. The G1 and G2+M peaks at time $t$ (relative to the cycle time) after synchronization can be obtained by distributing the proportions from Equations 1 and 3 (multiplied by the total counts in the experimental data) between the appropriate channels, according to the Gaussian spread of the G1 DNA fluorescence. The S-phase distribution is obtained by finding the "effective mean channel" of the distribution, EMS, which for the $I^{th}$ cycle is given by,

$$\mathrm{EMS(I)} = \mathrm{M} + \left[\frac{\mathrm{M}-1}{t_S}\left[t-(t_{G1}+\mathrm{J})\right]\right]$$

where $J=I-1$, M is the channel of the mean of the G1 DNA distribution and I varies from K through N cycles. It can be seen that $\mathrm{EMS}(1)=\mathrm{M}$ for $t=t_{G1}$, at which point half of the cells will have entered S. The shape of the distribution is computed with a channel variance, $S(I)^2$, given by,

$$S(I)^2 = \left[\frac{M-1}{t_S}\right]^2 (\sigma_{G1}^2+\sigma_S^2+(\sigma_{tc}^2\times J))$$

This accounts for the spreads in the G1 and S intermitotic phase times, and only that portion of the S distribution lying within the interval $M+1\rightarrow 2M-1$ channels inclusive represents cells in S, irrespective of the values of EMS(I) and S(I). A further broadening is now superimposed for the increase in the fluorescence standard deviation with increasing channel number, and the whole distribution is obtained by the appropriate summation.

It was shown previously (Watson, 1977) that the durations of the phases relative to the cycle time can be obtained by analysing the histogram of cells in asynchronous growth. This analysis will also give the standard deviation of the fluorescence of cells in G1. Thus, the number of parameters required to effect the analysis of synchronized populations is reduced to 4. These are: the 3 standard deviations of the intermitotic phase times, and the time from synchronization, $t$, relative to the cycle time. The computer program uses an iterative technique to find the "best fit" combination of values for these unknowns which gives the minimum sum of deviations between the experimental and synthetic data in each channel of the histogram. As the absolute times between synchronization and collection of data are known, the mean values for the cycle and intermitotic phase times, with their standard deviations can be calculated. The values of K and N are set depending on a guessed value of $t$, and they are reset by the programme if necessary. A subsidiary routine has also been written, which will analyse a number of histograms with common values for the respective phase time CVs at each time point. This takes the values of the CVs from the individual analyses, calculates the average and finds the common values which best fit all the data.

The durations of the intermitotic phase times relative to the cycle time were determined by analysing an asynchronous exponentially growing control population as described previously (Watson, 1977). This gave values of 0·36, 0·54 and 0·1 for $t_{G1}/t_c$, $t_S/t_c$ and $(t_{G2}+t_M)/t_c$ respectively, with a G1 DNA mean in Channel 30 and s.d. of 2·5 channels. The computed proportion in S phase was 49% which corresponded with a [$^3$H]TdR labelling index of 52%. Identically fixed and stained mouse thymocytes gave a peak in Channel 15 which was two channels wide at 50% of the maximum height. All subsequent histograms were recorded after any necessary adjustments had been made to the instrument to obtain identical control thymocyte distributions. A total of 15 DNA histograms was obtained following synchronization. These were analysed independently to obtain estimates of the phase coefficients of variation and of $t$, defined as the time from synchronization to the point in cycle time at which the data were obtained. Fig. 1 shows two values of $t$ plotted against each time point. These represent the maximum and minimum values that can produce a satisfactory simulation at each point irrespective of the estimates of the phase-time CVs. The line in Fig. 1 has been drawn to pass through the origin and within the possible range of $t$ at all times. The reciprocal of the slope gives a provisional cycle time estimate of 13·3 h. The phase-time CVs varied between 10% and 30% in the independent analyses and the mean values were 23%, 18% and 26% for $t_{G1}$, $t_S$ and $t_{G2}+t_M$ respectively.

Using these values as the starting point, all the experimental histograms were analysed with common values of the respective phase-time CVs which were varied in 2% increments. The cycle-time estimates were varied between 12·3 and 14·3 h in 0·5-h increments. This analysis produced the results given in the Table, and Fig. 2 shows the synthetic histograms fitted to the experimental data with these parameters. The uninterrupted curves bound the computed histograms, and the dotted curves bound the theoretical S-phase distributions. The points represent the experimental data, the number of cells in each channel corresponding to a given DNA content, and the times in hours from synchronization, are given on individual panels.

The computed proportions in S-phase at time $t$ are shown in Fig. 3 as the curve, with the $^3$H-TdR pulse-labelling indices represented by the points.

FIG. 1.—Computed fractions of the cell cycle time, $t$, plotted against the absolute times from synchronization. The maximum and minimum values of $t$ which gave a satisfactory simulation are shown at each time point. The line has been drawn to pass through the origin and within all the ranges of $t$.

DISCUSSION

Generally, the data shown in Fig. 2 are adequately fitted by the simulations generated with the parameters given in the Table, but there are some exceptions. The 10-h and 12-h computed histograms both tend to underestimate the proportion of G1 cells in the 2nd cycle, and to overestimate the proportions in S or G2+M of the 1st cycle. The individual analyses of these two points produced better fits when the CVs of $t_{G1}$ were 10% and 15% for 10 and 12 h respectively, and where the CV of $t_S$ was 25% for both. These individual best-fit CVs for $t_{G1}$ and $t_S$ are almost a reversal of the respective values of 23% and 16% which were found to be the best common values for all the histograms. The 24-h point shows a similar poor fit for the G1 peak at the start of the 3rd cycle. Similarly, these data were better fitted in the individual analysis where the CVs of $t_{G1}$ and $t_S$ were 25% and 20% respectively, both a little greater than the common values, and where the cycle time predicted from $t$ was less than 13·3 h. The 28-h point can also be fitted better with higher CVs.

Comparison of the computed proportions in S obtained from Fig. 2 with the

FIG. 2.—Synthetic histograms generated with the parameters given in the Table, continuous curves, fitted to the observed numbers of cells with a given DNA content. The dashed curves show the theoretical S-phase distributions the times in hours, from synchronization are shown on individual panels. The ordinate scales represent 250 cells per division.

FIG. 3.—Comparison of the pulse [$^3$H]TdR labelling indices, (the points), with the computed proportions in S (the curve generated from the parameters given in the Table).

[$^3$H]TdR labelling indices in Fig. 3 also shows some discrepancies. Apart from the 2-h point, all the labelling indices lie below the computed curve during the first wave, and both the 14-h and 28-h labelling indices are about 20% higher than the curve. The 10-h and 12-h experimental values are quite clearly overestimated by the model. This is compatible with the visual impression obtained from Fig. 2, where the DNA data also appear to be overestimated. The experimental data in Fig. 3 suggest a slightly shorter S phase than that predicted by the analysis of the control log-phase histogram data, and a higher CV for $t_{G1}$ or $t_S$. However, even allowing for these changes, the experimental data at 8 h 25% below the predicted value, would still be too low, and it appears to be an artefact.

These various discrepancies may be due to the assumptions in the model, the experimental technique or a combination of both. The most likely sources of error within the model are the choice of Gaussian-distributed mean phase times and the assumption of a linear DNA increase. Recent data obtained by Thilly, Arkin and Wogan (1977) in a synchronized HeLa system show a sigmoid increase in DNA with time. Data obtained for the synthesis rates in human bone marrow (Lajtha *et al.*, 1960) and for the time sequence of human chromosome duplication in cultured lymphocytes (Gilbert *et al.*, 1962) suggest that the increase in DNA is unlikely to be linear with time in these systems. It is possible that a better overall fit could be obtained with a different choice of distribution (*e.g.* log-normal) to describe the phase-time variation, and an assumed sigmoid function to describe the increase in DNA.

A further source of error could arise from the assumption that the mitotic selection procedure does not alter the relative phase durations. Values for these parameters were obtained from the completely unperturbed control log-phase DNA histogram and were applied to the analysis of the post-selection data. Although the synchronization procedure is more laborious than other methods (*e.g.* $G_1$ block produced by cyclic-AMP—Gray, 1976), it was felt that mitotic selection should give the tightest synchrony with the minimum of artefact. However, whilst care was taken to maintain the selected cells at 37 °C wherever possible, some cooling must have occurred during the centrifugation step, and it takes at least 15 min to get the selected cells back into flasks, gassed and in the incubator. Furthermore, the cell densities of the control log-phase and selected cell monolayers were not identical. All these factors may make slight differences to the relative post-selection phase times, which may not be accurately represented by the unperturbed control data.

Per cent labelled mitoses (PLM) data obtained previously (Twentyman *et al.*, 1975) indicate values within the ranges 0–2 h, 8–9 h and 1·5–2·5 h respectively for $t_{G1}$, $t_S$ and $t_{G2}$, with a median cycle time of 11–12 h. Although there was an interval of more than 2 years between the two sets of experiments (which may make comparisons a little artificial), the agreement between $t_S$ and $t_{G2}$ from the two methods is acceptable, but that for $t_{G1}$ is not. Furthermore, the cycle time was about 2 h longer in this analysis, and the computed proportion in S and labelling index were about 50% for the control log-phase cells in these studies, which is approximately 10% lower than reported previously (Twentyman *et al.*, 1975; Watson, 1977). Brooks (1976) has shown that the progression from G1 to S in 3T3 cells is dependent on "serum factors", and it has also been shown (Twentyman *et al.*, 1975) that EMT6/M/CC cells appear to effect changes in the medium very rapidly. We have noted, during a number of synchrony experiments, that the cycle time varies between 12 and 18 h. This is due, at least in part, to different serum batches, though cells in their second passage after removal from liquid $N_2$ tend to have the longest intermitotic intervals. It has also been noted that these cycle-time variations are mainly produced by changes in the G1 duration, as $t_S$ and $t_{G2}$ have always been fairly constant, with respective values within the ranges 7–9 h and 1·5–2·5 h. It is possible, therefore, that both the lower proportion in S and the increased cycle time in this analysis, compared with the values previously reported, could be due to a slightly lengthened G1 phase. If 2 h is subtracted from the $t_{G1}$ in the Table, we get values of 0·25, 0·64 and 0·11 for $t_{G1}/t_c$, $t_S/t_c$ and $t_{G2}/t_c$ respectively, and the proportion in S increases from 0·49 to 0·60. These values are almost identical to those given previously for the analysis of EMT6/M/CC cells during log-phase growth (Watson, 1977).

TABLE.—*Cell Cycle and Intermitotic Phase Times with their Standard Deviations*

| Phase | Relative duration ($t$) | Duration (h) | s.d. (h) | Coefficient of variation |
|---|---|---|---|---|
| G1 | 0·36 | 4·8 | 1·10 | 0·23 |
| S | 0·54 | 7·2 | 1·15 | 0·16 |
| G2+M | 0·10 | 1·3 | 0·36 | 0·28 |
| Whole Cycle | 1·00 | 13·3 | 1·64 | 0·12 |

Gray (1976) has also produced a model to analyse DNA histograms. This is based upon the Takahashi–Hogg–Mendelsohn model of the cell cycle (1971), which employs Gaussian-distributed mean phase times and has the added sophistication that a non-linear increase in DNA can be encompassed. However, Gray's method has a practical disadvantage which stems from the restraint that the phase-time CVs can only be varied in discrete steps. The step size is inversely proportional to the number of arbitrary subcompartments that a particular phase is divided into (Takahashi, 1966, 1968). Hence, to obtain low CVs a large number of phase subcompartments are needed, which in turn requires a similarly large number of differential equations to be solved simultaneously. Although Gray uses the fast "predictor-corrector" method of Hamming

(1973) to solve these equations, the computing time and central memory requirements must be considerable. By basing this model on the theory presented by Hartmann and Pederson (1970) a single analytic equation is obtained for each of the three cell-cycle phases. This represents a considerable simplification, with concomitant reduction in computing requirements, compared with any method based on the Takahashi–Hogg–Mendelsohn model.

In its present form, the model produces at least a reasonable approximation to the experimental data, but the possibility of a non-linear increase in DNA remains to be investigated further.

### REFERENCES

Brooks, R. F. (1976) Regulation of the Fibroblast Cell Cycle by Serum. *Nature, Lond.*, **260**, 248.

Crissman, H. A. & Steinkamp, J. A. (1973) Rapid, Simultaneous Measurement of DNA, Protein and Cell Volume in Single Cells from Large Mammalian Populations. *J. Cell Biol.*, **59**, 766.

Crissman, H. A. & Tobey, R. A. (1974) Cell-cycle Analysis in 20 Minutes. *Science, N.Y.*, **184**, 1297.

Gilbert, C. W., Muldal, S., Lajtha, L. G. & Rowley, J. (1962) Time-sequence of Human Chromosome Duplication. *Nature, Lond.*, **195**, 869.

Gray, J. W. (1976) Cell-cycle Analysis of Perturbed Cell Populations; Computer Simulations of Sequential DNA Distributions. *Cell & Tissue Kinet.*, **9**, 499.

Hamming, R. W. *Numerical Methods for Scientists and Engineers.* New York: McGraw-Hill.

Hartmann, N. R. & Pederson, T. (1970) Analysis of the Kinetics of Granulosa Cell Populations in the Mouse Ovary. *Cell & Tissue Kinet.*, **3**, 1.

Krishan, A. (1975) Rapid Flow Cytofluorimetric Analysis of Mammalian Cell Cycle by Propidium Iodide Staining. *J. Cell Biol.*, **66**, 188.

Lajtha, L. G., Oliver, R., Berry, R. J. & Hell, E. (1960) Analysis of Metabolic Rates at the Cellular Level. *Nature, Lond.*, **187**, 919.

Quastler, H. & Sherman, F. G. (1959) Cell Population Kinetics in the Intestinal Epithelium of the Mouse. *Expl. Cell Res.*, **17**, 420.

Rockwell, S. C., Kallman, R. F. & Fajardo, L. F. (1972) Characteristics of a Serially Transplanted Mouse Mammary Tumour and its Tissue Culture adapted Derivative. *J. natn. Cancer Inst.*, **49**, 735.

Takahashi, M. (1966) Theoretical Basis for Cell Cycle Analysis, I. *J. theoret. Biol.*, **13**, 202.

Takahashi, M. (1968) Theoretical Basis for Cell Cycle Analysis, II. *J. theoret. Biol.*, **18**, 195.

Takahashi, M., Hogg, J. & Mendelsohn, M. L. (1971) The Automated Analysis of FLM Curves. *Cell & Tissue Kinet.*, **4**, 505.

Terasima, T. & Tolmach, L. J. (1963) Growth and Nucleic Acid Synthesis in Synchronously Dividing Populations of HeLa Cells. *Expl. Cell Res.*, **30**, 344.

Thilly, W. G., Arkin, D. I. & Wogan, G. N. (1977) Nucleic Acid Content of HeLa S3 Cells during the Cell Cycle: Variations between Cycles. *Cell & Tissue Kinet.*, **10**, 81.

Trujillo, T. T. & van Dilla, M. A. (1972) Adaptation of the fluorescent Feulgen Reaction to Cells in Suspension for Flow Microfluorometry. *Acta cytol.*, **16**, 26.

Twentyman, P. R., Watson, J. V., Bleehen, N. M. & Rowles, P. M. (1975) Changes in Cell Proliferation Kinetics Occurring during the Life History of Monolayer Cultures of a Mouse Tumour Cell Line. *Cell & Tissue Kinet*, **8**, 41.

Watson, J. V. (1977) The Application of Age Distribution Theory in the Analysis of Cytofluorimetric DNA Histogram Data. *Cell & Tissue Kinet.*, **10**, 157.